# Environmental Isocyanate-Induced Asthma: Morphologic and Pathogenetic Aspects of an Increasing Occupational Disease

**DOI:** 10.3390/ijerph8093672

**Published:** 2011-09-09

**Authors:** Annette Fisseler-Eckhoff, Holger Bartsch, Rica Zinsky, Joachim Schirren

**Affiliations:** 1 Institute for Pathology and Cytology/Dr. Horst Schmidt Kliniken GmbH, Academic Hospital of the Johannes Gutenberg University, Mainz, Ludwig-Erhard-Str. 100, Wiesbaden 65199, Germany; E-Mails: Bartsch@pathologie-wiesbaden.de (H.B.); Zinsky@pathologie-wiesbaden.de (R.Z.); 2 Clinic of Thoracic Surgery/Dr. Horst Schmidt Kliniken GmbH, Academic Hospital of the Johannes Gutenberg University, Mainz, Ludwig-Erhard-Str. 100, Wiesbaden 65199, Germany; E-Mail: Joachim.Schirren@HSK-Wiesbaden.de

**Keywords:** occupational disease, isocyanate, occupational asthma, pathological findings, clinical findings, genetic predisposition

## Abstract

Occupational diseases affect more and more people every year. According to the International Labour Organization (ILO), in 2000 an estimated amount of at least 160 million people became ill as a result of occupational-related hazards or injuries. Globally, occupational deaths, diseases and injuries account for an estimated loss of 4% of the Gross Domestic Product. Important substances that are related to occupational diseases are isocyanates and their products. These substances, which are used in a lot of different industrial processes, are not only toxic and irritant, but also allergenic. Although the exposure to higher concentrations could be monitored and restricted by technical means, very low concentrations are difficult to monitor and may, over time, lead to allergic reactions in some workers, ending in an occupational disease. In order to prevent the people from sickening, the mechanisms underlying the disease, by patho-physiological and genetical means, have to be known and understood so that high risk groups and early signs in the development of an allergic reaction could be detected before the exposure to isocyanates leads to an occupational disease. Therefore, this paper reviews the so far known facts concerning the patho-physiologic appearance and mechanisms of isocyanate-associated toxic reactions and possible genetic involvement that might trigger the allergic reactions.

## 1. Introduction

Exposure to toxic or irritant substances at workplaces is a health risk and an increasing problem, concerning the affected persons on one side and the economic financial sources of the industrialized world on the other side. In 2000 alone, according to the International Labour Organization (ILO; www.ilo.org), at least 160 million people became ill as a result of occupationally-related hazards or injuries. Globally, occupational deaths, diseases and injuries account for an estimated loss of 4% of the Gross Domestic Product [1]. Taking only the cases of occupational asthma for the United Kingdom in the year 2003 into account, the costs are estimated to reach up to £100 million. More than half of this is caused by isocyanates [2].

Occupational asthma (OA) could be divided into a nonimmunological, irritant-induced asthma and an immunological, allergy-induced asthma [3–6], which will be addressed in this review. In addition, allergy-induced asthma can be caused by two different groups of agents: high molecular weight proteins (>5,000 Da) or low molecular weight agents (<5,000 Da), generally chemicals like the isocyanates [4–6], as will be discussed herein.

In occupational disease cases, the affected workers are forced to abandon their jobs, in the worst case forever or at least temporarily to recover. Therefore, it is of major concern to recognize possible occupational burden and their clinical and morphologic/pathologic signs as early as possible in order to avoid the exposure ending in a disease, as in the case of isocyanate-induced asthma, the symptoms persist even after cessation of the exposure [7,8]. For many substances dangerous and prescriptive limits have been implemented, but a lot of chemical substances are not only toxic or irritant, but could also lead to hypersensitivity reactions. The major problems are that: (1) these agents induce the hypersentitivity reactions already at very low concentrations, which technically are difficult to monitor, and (2) not all exposed workers are sensitive and develop the disease over time. All this together makes it difficult to obtain an effective prophylaxis if the underlying pathological mechanisms of this allergic reaction are not completely understood. Isocyanates and the large group of its derivatives belong to that group of substances which are important in the above mentioned context.

### Isocyanate Occurrence

Isocyanates are very reactive chemicals characterized by one or more isocyanate groups (–N=C=O). The main reactions of this chemical group are addition reactions with ethanol, resulting in urethanes, with amines (resulting in urea derivates) and with water. Here, the product is carbamic acid which is not stable and reacts further to amines, releasing free carbon dioxide. Diisocyanates and polyisocyanates are, together with the largely nontoxic polyol group, the basic building blocks of the polyurethane (PU) chemical industry, where they are used solely or in combination with solvents or additives in the production of adhesives, foams, elastomers, paintings, coatings and other materials.

Since the 1930s the usage of isocyanates has grown rapidly all over the World, reaching millions of tons that nowadays are produced and used annually in many different industries, *i.e.*, the car industry, aerospace industry, metal-working industry, wood-working industry, mining and many others [9,10]. Beside of their widespread industrial usage, isocyanates are also more and more used in private households, for example in paints or construction foam.

In generally isocyanates are used as oligo- and polymers. The route of exposure largely depends on workplace conditions, especially the given concentration and the temperature during the manufacturing process. The exposure occurs mainly by inhalation. Oral incorporation by smoking or eating at the workplace or skin exposure are also possible mechanisms [11,12].

The current legal safety level for isocyanate concentrations at workplaces in Germany varies between 0.024 mg/m^3^ for methyl isocyanate and up to 0.054 mg/m^3^ for dicyclohexylmethan-4,4′-diisocyanate, as listed in the attachment to the TRGS (Technische Regel für Gefahrstoffe) 430 [13]. In the United Kingdom the workplace exposure limit is 0.02 mg/m^3^ for all isocyanates for an 8 hour time average and 0.07 mg/m^3^ for a 15 minute short time exposure [14].

Diisocyanates at high concentrations can have direct toxic effects on mucous membranes [15] or can act at low concentrations as sensitizing agents after binding to different proteins. Concentrations of isocyanate as low as 1 ppm has been confirmed to induce significant functional changes in humans and inflammation processes in lung tissues [16]. In the UK there is a high incidence for OA, with isocyanates being the most common reason [17]. In Germany, more than 50,000 workers are exposed to low concentrations of diisocyanates. Between 5% and 25% of these workers may be expected to develop respiratory disorders [18–21]. As the exposure can happen in combination with different solvents and at different concentrations, the monitoring of workplaces might be difficult [10,22].

## 2. Clinical Manifestations after Exposure to Isocyanate

Clinical symptoms after chemical induced asthma are rarely seen shortly (meaning two to four hours) after exposure, but develop after a latency period of some weeks up to months [22]. Exposure to methylene diphenyldiisocyanate (MDI), hexamethylene diisocyanate (HDI), and toluene-2.4-diisocyanate (TDI) can lead to allergic reactions with striking similarities to allergic asthma (type 1) [10,23]. The resulting isocyanate asthma accounts for the leading causes of occupational induced asthma bronchial worldwide.

After sensitization even very small amounts of isocyanate can induce asthmatic reactions. This and other facts, including the aforementioned latency period, the reaction of only a part of the exposed workers or the delayed reaction strongly remind one of the classical allergic IgE mediated asthma. After exposure, up to 50% of the affected people present with specific IgE or IgG antibodies depending on the antigen used for antibody detection [10]. Up to now it has been a matter of intense debate, if isocyanate asthma really is IgE mediated or IgE independent [20,23,24].

### 2.1. Morphologic Manifestations after Isocyanate Exposure of the Lung

Morphological changes in lung epithelium after isocyanate exposure were described in humans (e.g., [8, 25–27]), cell cultures (e.g., [28,29]), and in experimental animal models (e.g., [30–34]). Cell culture analyses with human bronchial epithelium cells after exposure to 20 ppb toluene diisocyanate (TDI) demonstrate a disintegration of tight junctions between epithelial cells and an increased mucus secretion. No changes in the motile cilia of the airway epithelia were seen at this relatively low concentration. After incubation with increasing concentrations of TDI (100 ppb and 500 ppb) cumulative damage and impairment of the cilia activity and integrity was visible [28]. The functional impairment of the cilia was explained by an interaction between isocyanate and cellular filaments like tubulin [28].

Pathological diagnostic findings in isocyanate-induced asthma are mainly based on rare case reports. In low concentrations isocyanates could induce allergic reactions like acute asthma bronchial, hypersensitive pneumonia or acute extrinsic-allergic bronchiolar alveolitis (acute or sub-acute, 1 to 6 months after exposure). After persistent exposure to low concentrations (e.g., after 6 months) chronic obstructive lung diseases or, rarely, lung fibrosis could develop. In a fatal case of TDI induced asthma, the autopsy showed findings identical to those found in status asthmaticus, like excessive production of mucus, overinflated lungs, extensive epithelia desquamation, thickening of bronchial wall due to sub epithelial stroma oedema, and increased lymphocyte infiltration in the lamina propria [27,35]. In contrast to non-occupational asthma, the bronchial smooth muscle cells were hypertrophic and disarrayed [35,36].

Elevated levels of immune cells including eosinophils, CD45 positive cells (mostly mononuclear cells, only rare neutrophils) and mast cells were found in different compartments. [37–39]. Especially in the epithelium, the number of mast cells were significantly increased, most of them degranulated [27].

Elevated levels of induced immune cells, especially CD4 but also CD8 positive T cells and the TH2 cytokines IL4 and IL5 were demonstrated in biopsies and sputum, comparable to immunological processes seen in atopic asthma [8,22,40–42].

## 3. Pathogenesis of Isocyanate Asthma

Several studies in humans have addressed the inflammatory process underlying isocyanate-induced asthma. As for HMW-induced occupational asthma the pathogenetic process is equal to an IgE dependent mechanism, the pathophysiology of OA induced by low molecular weight agents as isocyanate is not well understood (e.g., [5, 6,40,41]).

Elevated levels of induced immune cells, especially CD4 but also CD8 positive T cells and of different cytokines like IL-1β, IL-4, IL-5, IL-6, IL-15 and TNF-α were demonstrated in biopsies, broncho alveolar lavage (BAL), and sputum of patients with isocyanate-induced asthma [8,39,40,42–44]. IFN-γ but no IL-5 or IL-13 expression was detected in human T-cell lines after exposure to HDI [45]. Other studies found a predominant activation of neutrophils [16,46,47] and an increase in myeloperoxidase and IL-8 after exposure to TDI supporting the neutrophil recruitment [47,48]. An increased MMP-9 level in TDI exposed patients was found [49,50] associated with a decrease in MMP-7 expression and a regression of T_H_-2 type inflammation [50]. In late reaction after BAL Zocca *et al.* found increased levels of the chemotactic active leukotriene B4 in patients after exposure to TDI [51]. The role of neuropeptides in TDI induced hyperreactivity has been investigated so far in animal models [52,53]. Scheerens *et al.* demonstrated the role of sensory neuropeptides, especially tachykinins, in the development of airway hyperresponsiveness in a TDI induced mouse model [52] and Mapp *et al.* showed comparable effects in guinea pigs [53].

Taken together, these results show a heterogenic picture of a T_H_1 controlled inflammation process (TNF-α, IL-1, IL-8, INF-γ), but are also conform to a T_H_2 triggered allergic process (IL-4, IL-5, IL-6). These results mirror the controversial discussion, already above mentioned, about the impact of IgE antibodies in isocyanate induced asthma. Maybe, this is because heterogenic patient collectives were used in the investigation of isocyanate induced asthma, with differences in the type of isocyanate exposure (TDI, MDI, HDI, mixtures with other sensitizers), with different concentration and varying periods of incubation at the workplace, different time points for the investigation, and so forth.

**Figure 1:**
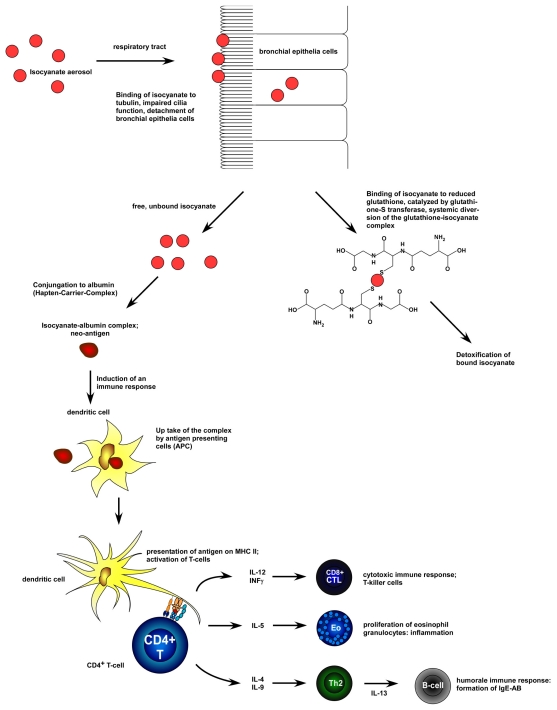
Predicted mechanism for the effects of isocyanate in the development of isocyanate induced asthma. The inhaled isocyanate or its derivates are absorbed by the bronchial epithelia cells, *i.e.*, by binding to tubulin. This results in a local disturbance and impairment of cilia function or a detachment of bronchial epithelia cells. Glutathione S transferase mediates the detoxification process. If the capacity of GSH is exhausted, the free isocyanate can bind to e.g., albumin. This isocyanate-albumin-complex acts as a neo-epitope, that is recognized by the immune system, and is leading to an induction of an immunological process by activation of CD4+ cells. The following inflammation process and, under certain circumstances, an additional induction of IgE antibodies induce the hypersensitivity reaction.

Incorporation of isocyanates is possible by pulmonary inhalation in the lungs, or skin exposure [12,54–56]. In particular isocyanates chemically react with NH_2_- and OH-groups of many proteins, as for example the already mentioned tubulin, albumin, creatin-18, and glutathione (GSH) [11,21,28,42,57,58]. GSH is a protective molecule defending cells from oxidative stress. The reduction of functional GSH caused by binding of isocyanate could lead to increasing damage caused by oxidative exposure, resulting finally in apoptosis of bronchial epithelial cells. This could explain the loss of functional epithelium seen in isocyanate induced asthma. Additionally the isocyanate-GSH complex is transported in the hole body during the detoxification process **[**22**]**. Furthermore, the reaction of isocyanates with proteins could lead to the formation of neo-antigens inducing an immunological reaction that might trigger an allergic asthma. Especially the binding to albumin seems to play a critical role in this process (Figure 1) [21,42,59,60].

Experiments in mice using high isocyanate doses demonstrate the presence of isocyanates and their derivatives in blood, gastrointestinal tract and in lower concentrations in other organs. Therefore, isocyanates are traceable throughout the body and are eliminated in the urine. In a monitoring of hydrolyzed urine samples for the presence of diamines, isocyanate exposure as low as 0.05 ppm could be detected in affected patients [10–12,61].

### 3.1. Influence of Genetic Factors in the Development of an Isocyanate Induced Asthma

As only 5% to 10% of exposed workers develop isocyanate-related asthma [62], the question arose whether a genetic predisposition plays a substantial role in the development of the disease. Several groups investigated possible candidate genes involved in isocyanate metabolism. Indeed, several polymorphisms in the involved genes were detected, and significant correlations to the development of isocyanate induced asthma could be demonstrated.

The already mentioned role of glutathione in the detoxification process of isocyanate strongly suggests that gene polymorphism coding for glutathione S-transferase (GST) play an important role in the efficiency of the elimination of isocyanates from the body and therefore might predict a susceptibility for the induction of an allergic reaction.

In a study by Piirilä *et al.* 182 workers exposed to different isocyanates (HDI, TDI and MDI) were screened for polymorphisms in the four glutathione S-transferase (GST) supergene family genes *GSTM1*, *GSTM3*, *GSTP1* and *GSTT1*, and 109 workers showed signs of isocyanate induced asthma, while 73 did not. The loss of the *GSTM1* gene (*GSTM1* null) was shown to be associated with a 1.89-fold increased risk to develop isocyanate-induced asthma. In addition, patients showing the *GSTM1* null genotype rarely generate IgE antibodies specific to isocyanate and show late reactions when tested in the specific inhalation challenge tests. The same late reaction was seen in patients with two alleles of the *GSTM A* genotype. In contrast, the genetic variant Val^105^ of the GSTP1 type in the homogenous constellation (GSTP1 Val/Val) was associated with high level of IgE antibodies. The frequency of the GSTP1 Val^105^/Val^105^ genotype had been 9.2% in the patient group and 6.8% in the control group [62]. In contrast, Mapp *et al*. found a decreased risk for isocyanate-induced OA in the GSTP1 Val^105^/Val^105^ genotype. In this study, 131 patients (92 asthmatic patients, 39 asymptomatic workers), all exposed to TDI, were examined. The frequency of the GSTP1 Val^105^/Val^105^ genotype had been 6.5% in the asthmatic group and 10.3% in the non-asthmatic group. The authors found an increase in the Val^105^/Val^105^ frequency up to 18.5% in the non-asthmatic group in individuals exposed to TDI for longer than 10 years, so the GSTP1 Val^105^/Val^105^ genotype showed a protective effect [63]. The same result was shown in another study performed by this group on 202 individuals [64]. Here it was shown that the GSTP1 Val^105^/Val^105^ genotype was associated with a decreased severity of bronchial hyperresponsiveness and lower level of IgE antibodies defined by positive skin test. The authors critically discussed, as the number of individuals with GSTP1 Val^105^/Val^105^ genotype in this cohort was small (n = 13), the findings should be confirmed in larger study samples [64]. As already mentioned by Mapp *et al.*, the contrary results in the studies of Mapp *et al.* and Piirilä *et al.* could be due to exposure to different isocyanates (TDI by Mapp *et al.*, HDI, MDI and TDI by Piirilä *et al.*), differences in the definition of asthma, bronchial hyperresponsiveness and atopy in different centers, differences in the level and duration of exposure, or the small allele frequency in the cohorts.

The influence of these polymorphisms on the metabolism of the detoxification process of isocyanate was investigated in studies done by Broberg *et al.* They measured the concentrations of TDI in the air and those of the TDI metabolites 2,4- and 2,6-toluenediamine (TDA) in urine samples and plasma, and determined the corresponding genotype. Those who were homozygotic for the GSTP1 Val/Val allele had the highest regression of 2,4-TDA in plasma and therefore the highest concentration in urine when compared to GSTP1 Val/Ile and Ile/Ile. Similar results were obtained for 2,6-TDA. Different genotypes of the genes *GSTM1*, *GSTP1*, *GSTT1*, CYP1A1*2A and *MPO* also influenced the ratio between TDA in plasma and urine and therefore could predict a higher sensibility for isocyanate induced asthma in affected patients. In accordance with Mapp *et al.* [63] the authors found a tendency towards a protective effect of the GSTP1 Val^105^/Val^105^ genotype [65,66]. The mechanism by which this GSTP1 Val^105^/Val^105^ genotype variant influences the TDI metabolism is not known. Broberg *et al.* suggested a more effective conjugation of TDI to the GSTP1 Val^105^/Val^105^ variant, therefore reducing the concentration of unbound TDI in the plasma, which would then on the other hand result in lesser TDI-protein conjugates for allergic reactions [66].

IgE-independent isocyanate asthma could be caused by cell mediated immunity as described by Mapp *et al.* [24]. As a sign of this process many eosinophile granulocytes and activated T-cells are detectable in the bronchial mucosa. Important for the activation of T-cells is their interaction with antigen presenting cells (APCs). These cells (including dendritic cells, monocytes, macrophages and B-cells) present the antigen in association with the MHC II complex. This complex is encoded by the HLA (human leukocyte antigen) genes. Therefore, it is reasonable, that polymorphisms in the HLA genes influence the susceptibility for isocyanate asthma. In the publication of Mapp *et al.*, 67 asthmatic patients and 27 non asthmatic, but also TDI exposed workers were included in the study. It was shown, that the HLA class II genotypes DQA1*0104 and DQB*0503 are significantly overrepresented in affected persons, whereas the genotypes DQA1*0101 and DQB1*0501 were significantly overrepresented in exposed, but asymptomatic workers. On the other hand, the *DRB1* genotype did not show any influence [67]. Interestingly, another investigation that involved the same polymorphisms in the HLA II genes by Rihs *et al.* found no correlation [68]. This discrepancy might be explained by the usage of different isocyanates (TDI, HDI, MDI) by Rihs instead of only one (TDI) used by Mapp, and by using healthy, not exposed blood donors as a control panel.

In a recently published investigation Choi *et al.* aimed to clarify the influence of HLA I and HLA II gene polymorphisms on the risk to develop diisocyanate-induced occupational asthma [69]. Two hundred and fifty eight (258) people were enrolled in this study, 84 patients showed a diisocyanate induced asthma, 47 were exposed controls and 127 people served as non exposed controls. Here the haplotype HLA DRB1*1501-DQB1*0602-DPB1*0501 was detected significantly more often in TDI-OA-Patients then in all other control patients. In addition, in patients with the DQB1*0402 allele IgE antibodies against TDI-albumin conjugates were more often found than in other groups. As these investigations were carried out in a Korean collective, it is not clear whether the results can be transcribed to a Caucasian collective, although the negative association for HLA class I genes has also been found in a Caucasian study cohort [70].

Beside HLA and GST gene polymorphism other genetic variations might be responsible for the susceptibility towards isocyanate-induced asthma. Kim *et al.* found an association of different genetic polymorphism in the region of the catenin alpha 3 (*CTNNA3*) gene with a TDI induced asthma phenotype. In this investigation 84 patients with TDI induced asthma and 263 unexposed controls were analyzed in a microchip based SNP analysis [71]. *CTNNA3* together with *CTNNA1* are coding for α-catenin, a key molecule in the E-cadherin mediated cell-cell adhesion complex.

The collective investigated already by Piirilä for *GST* gene polymorphisms was also tested for polymorphisms in the N-acetyltransferase (NAT) genes. In the *NAT1* genotype (presumably a slow acetylating phenotype) the risk for the development of isocyanate-induced asthma was increased [72].

Taking these demonstrated genetic polymorphisms, which could be associated with isocyanate induced asthma phenotype, into account, the question arose whether these genotypes play a role in the prevention and appraisal of occupational asthma.

Bernstein *et al*. investigated the effect of genetic nucleotide polymorphisms of interleukin 4 receptor alpha (*IL4RA*), *IL-13*, and *CD14* in isocyanate exposed workers and found a statistically significant association of diisocyanate asthma with the *IL4RA* (I50V), *IL13* (R110Q), and *CD14* (C159T) genotype combinations, but only in HDI exposed workers and not in those exposed to MDI or TDI. As discussed by the authors, the reason for this finding might be a statistical artifact due to the greater number of HDI-exposed subjects available in these studies [73,74].

## 4. Diagnosis of Occupational Isocyanate Induced Asthma

In Germany, occupational diseases concerning contact with isocyanate (with the exception of skin effects) are summarized under the BK number 1315. At this time, approximately 50 new cases are accepted as occupationally-induced every year [12]. Due to the variety of symptoms, the variable exposure including not only isocyanate, but also other solvents and the so far not complete known pathological mechanisms, the clinical diagnosis is still not an easy call and needs a multistage diagnostic approach (Table 1) [22,75].

After the clinical confirmation of an asthmatic disease, the next step is to review the occupational history and the possible exposure to isocyanate or its derivatives. Thereafter, the specific inhalation challenge test (SIC) is the reference standard. Here, the direct correlation to the asthma inducing substance could be resolved [6,76]. Unfortunately, this test needs special equipment and trained personal, and for this reason it is only available in a small number of laboratories [77]. The test is able to mimic the occupational load in a defined and nearly realistic way, but in some cases it could take up to several days, and even though it is time consuming and expensive, there have been reports of false negative results [76]. Especially after cessation of exposure to isocyanates the result of a first SIC may be negative. In a study of Sastre *et al.*, 19 patients were challenged with TDI. Overall 16 showed an allergic reaction, three of them (16%) where false negative in the first SIC, but positive in a second SIC [78]. A recently published technical improvement might enhance the SIC test [79].

Other diagnostic possibilities include the use of spirometry and the evaluation of the peak expiratory flow rate (PERF). In both cases changes could be evaluated in a working period in comparison to a working free time period (weekend, holiday). The observation period should last at least four weeks, including one week free of occupational charge. During this period PERFs should be monitored four times a day [76]. When compared to the reference standard (SIC), the work attendant PERF showed a sensitivity of 64% and a specificity of 77% [6].

Although the experimental findings concerning the significance of the presence of IgE-antibodies in isocyanate-induced asthma are still very controversial, the immunological detection of isocyanate specific IgE or IgG antibodies are an alternative diagnostic tool. A positive test for IgG antibodies clearly proves an isocyanate exposure [20,23,24] and the detection of IgE antibodies is a very high predictor for isocyanate-induced asthma [10,19,21]. Even though the sensitivity is low (21% to 55% are described, depending on the antigen used) the specificity is between 89% and 100% [76]. In a follow up study over 16 years Piirilä *et al.* showed a better clinical outcome for IgE positive patients when compared to IgE negative [80]. Because of these problems, the search for reliable biomarkers is ongoing, as nicely summarized in a recently published paper of Palikhe *et al.* [81].

## 5. Future Prospects

Jobs associated with contact to isocyanates are steadily increasing. In the same way, the number of cases showing occupational asthma is growing. Therefore the diagnosis of occupational induced Isocyanate asthma has a very high relevance due to the legal consequences. The so far described morphologic subsumable changes could be seen also in other inflammatorily or toxically induced damage of the lung. Therefore there are no specific or characteristic morphologic changes for isocyanate-induced asthmatic disease known to date. Hence, beside the pathological and anatomical diagnostic, the clinical anamnesis must include data for the occupational burden of isocyanate in order to classify the manifested asthma as occupationally induced.

In this context it has to be emphasized that only in rare cases histological specimens are available when isocyanate-induced asthma is diagnosed. Thus, it seems reasonable to enhance the sampling in order to search for isocyanate-induced asthma specific change(s) (on a morphological, immune histological or molecular basis) in order to be able to make an unequivocal call for an occupational disease.

## Figures and Tables

**Table 1:** Diagnostic tools for the conformation of an occupational isocyanate associated asthma [22], modified.

Diagnostic Approach	Result

Medical examination	
Occupational anamnesis, exposure on the job	Clinical verification of the asthmatic disease Confirmation of isocyanate exposure: characterization of the chemical substances Specification of the grade of exposure

Physiological tests:	
Metacholine provocation test Spirometry PERFs (peak expiratory flow rate)	Confirmation of the diagnose “asthma” and documentation of the occupational causation
SIC (specific inhalation challenge)	“Diagnostic reference standard”, characterization of the specific substance

Immunological investigations	
Isocyanate specific IgE	Strong indicator for isocyanate induced asthma, not very sensitive
Isocyanate specific IgG	Conformation of exposure towards isocyanate
